# What are they all doing in that restaurant? Perspectives on the use of theory of mind

**DOI:** 10.3389/fpsyg.2024.1507298

**Published:** 2024-12-23

**Authors:** Ilaria Gabbatore, Francesca M. Bosco, Maurizio Tirassa

**Affiliations:** ^1^Department of Humanities, GIPSI Research Group, University of Turin, Turin, Italy; ^2^Department of Psychology, GIPSI Research Group, University of Turin, Turin, Italy; ^3^Neuroscience Institute of Turin, University of Turin, Turin, Italy; ^4^Department of Psychology, University of Turin, Turin, Italy

**Keywords:** theory of mind, social cognition, mental states, assessment tools, Th.o.m.a.s.

## Abstract

If “theory of mind” is conceived as reasoning in a strict sense, then it can be said to be useful only at certain times; however, this leaves the rest of social cognition hardly comprehensible. If “theory of mind” is used instead to refer to a mentalist ontology and the consequent awareness that we ourselves and the others function on mental states, then we need new approaches that explain the flow of social experience. To illustrate these points, we outline the general conceptual framework that underlies most empirical studies of theory of mind and discuss their pros and cons; then, we discuss the Theory of Mind Assessment Scale, a tool developed to investigate the complexity of theory of mind, which adopts a different perspective and has been successfully tested on numerous populations.

## 1 Introduction

Most research on theory of mind participates in a common framework. The overall goal is to map the development of such faculty in the infancy and the childhood. Legitimate questions could be: what is an agent to a child? What kinds of entities do children perceive as agents, how, and why? What kinds of mental states and reasoning do children attribute to an agent, and through what kinds of reasoning of their own? Around what ages and through what steps do these developments take place? To find answers to these and other similar questions, different types of cognitive challenges are presented to children of different ages, either as problems explicitly posed by the experimenters or embedded in manufactured world situations that hopefully appeal to their spontaneous curiosity or desires. The responses or behaviors collected (whichever is required in each setting) are expected to provide information about the children’s naturally emerging social cognition. The ideal challenge is one which, by the very fact of being solved, proves beyond reasonable doubt the presence of the relevant form of psychological reasoning.

Of course this is a sensible strategy, for historical and conceptual reasons as well as for applicative ones. Studying the early development of a faculty, especially one that is so crucial in ontogeny and phylogeny, may help shed light on what the child’s cognitive endowment is before culture and individual experience become too important (Gabbatore et al., 2023). This research thus bears on the debate about nativism and the nature/nurture relationships and its more recent incarnation, namely the one about initial knowledge (e.g., Baillargeon et al., 2016). To understand the acquisition of theory of mind may also have important clinical correlates (especially for autism, which has been characterized as involving an impairment of theory of mind: Baron-Cohen, 1995; Happé and Frith, 1996) and pedagogical and educational applications (e.g., Grover, 2015; Lecce and Devine, 2022; Smogorzewska et al., 2020; Wang, 2015).

Because of this complex backdrop the goal, on the epistemological level and therefore on the methodological one, is to achieve the greatest possible clarity about what is going on in the child’s mind. This is also true of the study of theory of mind in animals, which was the actual field of Premack and Woodruff’s (1978) foundational article. The problem of how to distinguish between a “true” theory of mind and a “mere” expectation about another agent’s behavior was immediately raised in Dennett’s (1978) commentary on Premack and Woodruff’s paper. Dennett argued that certainty about someone’s capability of psychological reasoning can only be achieved if she can hold a negative belief about another agent’s knowledge, not a positive one. In other words, if Tommy and I have identical knowledge of a certain state of affairs it will not be clear to an observer whether I interpret and predict Tommy’s actions on the basis of his knowledge or mine; if, however, there is a knowable difference between Tommy’s knowledge and mine it will be possible to draw such distinction, thus proving whether I am aware that he has mental states of his own which need not be identical to mine.

This line of reasoning also provided the basis for the famous papers by Baron-Cohen et al. (1985) about autism, whose title of course echoed that of Premack and Woodruff, and by Wimmer and Perner (1983), who devised the first false belief task to be employed with young children.

The rest, as they say, is history: the false belief task has had its ups and downs, other experimental paradigms have been devised, theories have been proposed and refined (see, e.g., Kulke et al., 2019; Onishi and Baillargeon, 2005), but the general research framework has not changed much; nor, given its apparent overall reasonableness, have there been particularly compelling reasons to change it. We do not have the space to discuss this rich area here; excellent reviews and systematizations have been published by Barone et al. (2019), Matthews et al. (2018), Poulin-Dubois et al. (2023), Schneider et al. (2017), and Wellman et al. (2001).

## 2 Beyond childhood

The framework we have outlined has proven precious both in developmental and in clinical psychology. It is useful in general for locating specific turning points in the development or the decay of theory of mind; however, unlike what happens in geometry, such points do not allow to extrapolate a curve, nor do they tell much about the actual nature and functioning of theory of mind. When a child passes a certain task, all we know is that she possesses (and uses) the ability to do so. This is clearly important and interesting, but does not exhaust the questions: what does the child do when she is not handling false beliefs? What actually is her theory of mind and how does she use it in her everyday life? What becomes of her theory of mind as she grows to be an adolescent, an adult, and an elderly person? What are the workings of social cognition in the human species? The capability of passing an experimental task does not smoothly translate into the capability of interacting in real-life social situations, both because each kind of activity embodies different cognitive demands and because of the roles that are possibly played by motivation, social status, and other contextual factors (Astington, 2003; Massaro and Castelli, 2009).

Furthermore, except in particular clinical contexts, the tasks and experiments suitable for young children lose much of their usefulness at different ages. Most of them, if proposed to an elder child or an adult, would have him think that the experimenter was making fun of him or that there was some hidden trick. Even a serious response would not be informative anyway: once someone has started passing a task, he will probably just continue to pass it for the rest of his life.

Subtler, more naturalistic tasks have therefore been designed for the study of the adolescent and the adult theory of mind. Some, like the Reading the Mind in The Eyes (Baron-Cohen et al., 2001), assess specific abilities and thus maintain the “punctiform” approach that characterizes children tasks; others, like the Faux Pas (Stone et al., 1998) or the Strange Stories (Happé, 1994) tasks, explore the participant’s ability to handle the mental states surrounding some social mistake or blunder made by the fictional protagonists of short narratives. All focus on how the participants make sense of a scene of which they are spectators; while the specifics change from instance to instance, the underlying idea remains the same.

Other tools have been developed that focus on specific tasks or rely on video instead of narrative material, e.g., the Theory of Mind Picture Stories Task (Brüne, 2003), the Conversations and Intimations task (Ouellet et al., 2010), and the Virtual Assessment of Mentalising Ability (Canty et al., 2017). Karmakar and Dogra (2019) offer a review of the several tools available.

The Theory of Mind Assessment Scale (Th.o.m.a.s.; Bosco et al., 2016; Bosco et al., 2009a; Bosco et al., 2006), to whose development and application we and other colleagues have collaborated, takes a different stance. It consists in a semi-structured interview composed of 35–40 questions, lasting possibly around an hour. The number of questions is not rigid because if the interviewee spontaneously extends an answer to the contents of another question, the latter may be omitted. On the theoretical level, this tool views theory of mind as a complex, sophisticated faculty that humans employ for comprehending both a partner’s mental states and those of their own and for planning an attempt to modify them (see also Bosco et al., 2009b). The capability of affecting the interlocutors’ mental states is the foundation of human interactions (Tirassa, 1999; Tirassa and Bosco, 2008) and therefore requires first the capability of understanding what such states are, how they function, how they causally relate to each other and to the world, and what may affect them. It also requires to be able to distinguish the nature and functional role of at least a few basic mental states like beliefs, desires, intentions, or emotions. The interview explores all these aspects of theory of mind, gently pushing the interviewee to make explicit her awareness of the various issues involved. The transcript of the interview is assessed separately by two trained, independent judges on an established set of criteria; once any differences in assessment have been resolved, the final report provides a complex profile of the interviewee’s theory of mind. The Th.o.m.a.s. thus embodies a theory of theory of mind which goes beyond the punctiform measurement of a single one of its component abilities.

Initially developed in Italian (Bosco et al., 2006), the Th.o.m.a.s. was translated into English (Bosco et al., 2016), validated (Bosco et al., 2016) and successfully employed with populations such as typically developing (Bosco et al., 2014b) and self-injury adolescents (Laghi et al., 2016), sex offenders (Castellino et al., 2011), young women with bulimia nervosa (Laghi et al., 2014), persons with schizophrenia (Bosco et al., 2009a), with congenital heart disease (Chiavarino et al., 2015), alcohol use disorder (Bosco et al., 2014a), border personality disorder (BPD; Colle et al., 2019), opiate dependency (Gandolphe et al., 2018), persons receiving treatment for non-psychotic disorders (Francesconi et al., 2016), and persons with medication-overuse headache and migraine (Romozzi et al., 2022). In such populations the Th.o.m.a.s. has allowed to highlight profiles of theory of mind impairment (e.g., Bosco et al., 2024). For example, individuals with bulimia nervosa (Laghi et al., 2014) found it harder to accurately answer Th.o.m.a.s. questions that asked to reflect on other persons’ mental state (i.e., third-person ToM). In contrast, they found it easier to reason about their own mental states (i.e., first-person ToM). A similar pattern was identified in individuals with alcohol use disorder (Bosco et al., 2014a). In a related vein, people with borderline personality disorder (Colle et al., 2019) exhibited difficulties in Th.o.m.a.s. scales that evaluate the ability to attribute mental states from an allocentric perspective, i.e., one that is independent of one’s own standpoint. However, they performed similarly to controls on the scales based on the egocentric perspective. Interestingly, this discrepancy between allocentric and egocentric mindreading abilities was not observed in persons with a diagnosis of schizophrenia (Bosco et al., 2009a), who performed equally poorly as healthy controls on the Th.o.m.a.s. scales assessing these perspectives.

To avoid oversimplifying these results here, we refer interested readers to the specific papers for more details on the pattern of strengths and weaknesses across the various populations mentioned.

## 3 Humans as full-time mentalists

The Th.o.m.a.s. investigates the interviewee’s retrospective awareness of her theory of mind as it is generated at the time of the interview and stimulated by the interview itself. A brief discussion may be necessary.

Consider: as I chat idly with an old friend in a pub, do I need to engage in any reasoning to understand what she is saying? There is an obvious sense in which I (mostly) do not; yet, it is equally obvious that I am not viewing her (and the other patrons, the staff, and myself) like a behaviorist would want me to (Skinner, 1938; Watson, 1913), nor am I oscillating between behaviorism and mentalism or finding myself on some middle ground between the two. I just know my friend’s character, the general lines and many details of her life, her way of thinking and so on, and I interact with her accordingly. My comprehension of what she says comes in fully psychological terms even though I am not specifically reasoning about her mental states, or even wondering what they might be. Yet, I can always ask myself, more or less intensely, how she really feels about a certain matter or what the intents could be of a common acquaintance she is telling me about. In doing or not doing so, or doing so to a certain depth, I do not become more or less mentalist: I always am, but I dedicate variable amounts of time, attention and effort to actually reasoning about her thoughts, depending on how I sense the situation. This is only a matter of circumstances: it has nothing to do with having theory of mind or not or being a behaviorist; it has to do with the ways, the extent, the goals etc. in which I am using my knowledge of the mind.

This is just an instance of how consciousness and the mind always work. When I walk in the street I do not usually reason about colors; yet I see the world colored. I can pay cursory attention to the traffic lights: this is not really reasoning about them, it is just a slightly higher level of attention than I generally pay to the colors of the dress of the people I pass. I can engage in actual reasoning, e.g., when I start looking at the clothes in a shop, wondering what will go best with a certain dress I have at home. This may even become difficult, e.g., if there is not much light and I try to realize what color a certain dress really is. Yet, nobody would suggest that, when I am not reasoning about colors, I only see in black and white or in shades of grey.

The same applies to action. The extent to which our movements or speech are conscious and deliberate depends on what we are doing and why. When it is my turn to tell my friend what I have been up to since we last saw each other, I will probably not painstakingly choose each and every word to pronounce: I will just follow the thread of the conversation, taking care of the general sense of it and counting on her to understand it. However, if the topic shifts to something that I know troubles her, suddenly I will become much more careful about the possible effects of my words.

Any number of examples could be made. When I pass along the window of a restaurant, do I (normally) reason about what might have pushed all those people to get in or will I just take it for granted that they are hungry? Yet hunger is undoubtedly a mental state. Once again, my theory of mind is just revved down, so to speak, but never turned off, and always ready to return to full operation.

This is obvious in everyday life, but hard to capture in theoretical terms. Yet, we believe this point is crucial for the cognitive sciences in general and for this debate in particular. Not all that is mental is reasoning; not all that is mental is problem solving.

In retrospect, however, we are typically capable of summarizing sequences of events or thoughts and giving an average assessment of the quality and depth of our own and others’ performance as well as of the underlying cognitive framework and the results achieved.

## 4 Conclusion

To test a subject’s capability of handling one or more well-structured theory of mind problems does certainly say something. It is the only way we can achieve certainty of the presence of theory of mind in the cognitive architecture of other species. In ours, it may be useful for diagnostic purposes, if a theory exists that appropriately links those measures to the condition investigated, a bit like it is done with glycaemia and diabetes. For the same reasons and under the same conditions, it may help map the faculty’s development during childhood or its decay under specific conditions.

However, this strategy may be less informative about other issues. It has nothing to say about the social cognition of an agent who does not pass the relevant tasks, and is generally unable to provide a wider description of the workings of theory of mind. Some persons might pass all the tasks and still fail to decipher their spouse’s thoughts or to realize that they are being deceived in everyday circumstances; conversely, a child might get by just fine in everyday social life but struggle to solve the abstract problems. To equate theory of mind with the ability to pass certain chosen tasks also compels to lower as much as possible the age at which children become able to do so, both because that is the only description available of their social competence and because there is an implication that a child who does not solve the tasks can only be a behaviorist, which there are several reasons not to accept (Bosco and Tirassa, 1998). This is true of young children in general (Tirassa et al., 2006a) and in particular of the autistic ones, whose differences to typically developing children and subsequent possible development of a functioning social life become essentially incomprehensible.

This strategy may also prevent the exploration of other possibilities both for infant cognition (based, e.g., on intersubjectivity: Airenti, 2015; Trevarthen, 1998, or on a basic notion of sharedness: Tirassa et al., 2006a, 2006b) and for adolescence (e.g., Brizio et al., 2015). On a more contingent level, many existing tasks appear to favor an individualistic and spectatorial approach over one of sharing and participation and to limit the definition of theory of mind to the comprehension of an observed problem. However, theory of mind is much more than this, and even comprehension is more radically based in interaction than in mere observation (Trevarthen, 1998).

Therefore, it may be desirable to explore how people construe, describe and criticize their own theory of mind and that of the others, how they use it to capture and understand the causal relationships between mental states and between mental states and the world, and how they practically employ such knowledge to achieve actual changes in a given situation. This is what the Th.o.m.a.s. does. On the other hand, it requires the interviewee to be capable of sustaining the interview and to possess at least a working level of social awareness and expertise. Thus, until tools are developed that capture the best of the two worlds, a trade-off appears to exist between different approaches to the matter.

Since it functions in retrospect, leveraging on the interviewees’ recapitulation of their past experience, the Th.o.m.a.s. also avoids reducing social life to a sequence of formal problems to observe, reason about, and solve, interspersed with intervals that either remain incomprehensible or can only be described as behavioristic. Humans experience their social life as a continuous flow of thoughts, actions and events, always based on a mentalist ontology, whose workings include occasional bouts of actual reasoning when needed, with variable degrees of commitment and difficulty (and variable success). This special issue asks *when and how theory of mind is useful*. We believe that the answer depends less on how human beings function than on how theory of mind is defined. If theory of mind is conceived as reasoning in a strict sense, then it can be said to be useful only at certain times; this, however, leaves the rest of social life hardly comprehensible. However, if theory of mind is used to refer to a continuous mentalist ontology and the consequent awareness that we ourselves and the others function on mental states, then we need new approaches to study, describe and explain the flow of social experience and the ways in which we treat the problems that occasionally surface from it. In the meantime, there exists at least one instrument that allows a thorough exploration of the matter.

## Data Availability

The original contributions presented in the study are included in the article/supplementary material, further inquiries can be directed to the corresponding author.

## References

[ref1] Airenti G. (2015). Theory of mind: a new perspective on the puzzle of belief ascription. Front. Psychol. 6:1184. doi: 10.3389/fpsyg.2015.01184, PMID: 26321995 PMC4536386

[ref2] Astington J. W. (2003). “Sometimes necessary, never sufficient: false belief understanding and social competence” in Individual differences in theory of mind: Implications for typical and atypical development. eds. Repacholi B. Slaughter V. (New York, NY: Psychology Press), 13–38.

[ref3] Baillargeon R. Scott R. M. Bian L. (2016). Psychological reasoning in infancy. Annu. Rev. Psychol. 67, 159–186. doi: 10.1146/annurev-psych-010213-115033, PMID: 26393869

[ref4] Baron-Cohen S. (1995). Mindblindness. Cambridge, MA: MIT Press.

[ref5] Baron-Cohen S. Leslie A. M. Frith U. (1985). Does the autistic child have a “theory of mind”? Cognition 21, 37–46. doi: 10.1016/0010-0277(85)90022-8 2934210

[ref6] Baron-Cohen S. Wheelwright S. Hill J. Raste Y. Plumb I. (2001). The “‘Reading the mind in the eyes’” test revised version: a study with Normal adults, and adults with Asperger syndrome or high-functioning autism. J. Child Psychol. Psychiatry Allied Discip. 42, 241–251. doi: 10.1111/1469-7610.00715 11280420

[ref7] Barone P. Corradi G. Gomila A. (2019). Infants’ performance in spontaneous-response false belief tasks: a review and meta-analysis. Infant Behav. Dev. 57:101350. doi: 10.1016/j.infbeh.2019.101350, PMID: 31445431

[ref8] Bosco F. M. Capozzi F. Colle L. Marostica P. Tirassa M. (2014a). Theory of mind deficit in subjects with alcohol use disorder: an analysis of mindreading processes. Alcohol Alcohol. 49, 299–307. doi: 10.1093/alcalc/agt148, PMID: 24064369

[ref9] Bosco F. M. Colle L. De Fazio S. Bono A. Ruberti S. Tirassa M. (2009a). Th.O.M.A.S.: an exploratory assessment of theory of mind in schizophrenic subjects. Conscious. Cogn. 18, 306–319. doi: 10.1016/j.concog.2008.06.006, PMID: 18667334

[ref10] Bosco F. M. Colle L. Pecorara R. S. Tirassa M. (2006). ThOMAS, Theory of Mind Assessment Scale: uno strumento per la valutazione della teoria della mente. Sistemi Intelligenti XVIII, 215–242. doi: 10.1422/22502

[ref11] Bosco F. M. Colle L. Salvini R. Gabbatore I. (2024). A machine-learning approach to investigating the complexity of theory of mind in individuals with schizophrenia. Heliyon 10:e30693. doi: 10.1016/j.heliyon.2024.e30693, PMID: 38756573 PMC11096895

[ref12] Bosco F. M. Colle L. Tirassa M. (2009b). The complexity of theory of mind. Conscious. Cognit. 18, 323–324. doi: 10.1016/j.concog.2008.12.007 18667334

[ref13] Bosco F. M. Gabbatore I. Tirassa M. (2014b). A broad assessment of theory of mind in adolescence: the complexity of mindreading. Conscious. Cogn. 24, 84–97. doi: 10.1016/J.CONCOG.2014.01.003, PMID: 24491434

[ref14] Bosco F. M. Gabbatore I. Tirassa M. Testa S. (2016). Psychometric properties of the theory of mind assessment scale in a sample of adolescents and adults. Front. Psychol. 7:566. doi: 10.3389/FPSYG.2016.00566/BIBTEX 27242563 PMC4860419

[ref15] Bosco F. M. Tirassa M. (1998). “Sharedness as an innate basis for communication in the infant” in Proceedings of the 20th annual conference of the cognitive science society. eds. Gernsbacher M. A. Derry S. J. (Mahwah, NJ: Erlbaum), 162–166.

[ref16] Brizio A. Gabbatore I. Tirassa M. Bosco F. M. (2015). “No more a child, not yet an adult”: studying social cognition in adolescence. Front. Psychol. 6:1011. doi: 10.3389/fpsyg.2015.01011, PMID: 26347664 PMC4543799

[ref17] Brüne M. (2003). Theory of mind and the role of IQ in chronic disorganized schizophrenia. Schizophr. Res. 60, 57–64. doi: 10.1016/S0920-9964(02)00162-7, PMID: 12505138

[ref9031] Canty A. L. Neumann D. L. Fleming J. Shum D. H. (2017). Evaluation of a newly developed measure of theory of mind: the virtual assessment of mentalising ability. Neuropsychol. Rehab. 27, 834–870. doi: 10.1080/09602011.2015.1052820 26095322

[ref18] Castellino N. Bosco F. M. Marshall W. L. Marshall L. E. Veglia F. (2011). Mindreading abilities in sexual offenders: an analysis of theory of mind processes. Conscious. Cogn. 20, 1612–1624. doi: 10.1016/j.concog.2011.08.011, PMID: 21924641

[ref19] Chiavarino C. Bianchino C. Brach-Prever S. Riggi C. Palumbo L. Bara B. G.. (2015). Theory of mind deficit in adult patients with congenital heart disease. J. Health Psychol. 20, 1253–1262. doi: 10.1177/1359105313510337, PMID: 24287802

[ref20] Colle L. Gabbatore I. Riberi E. Borroz E. Bosco F. M. Keller R. (2019). Mindreading abilities and borderline personality disorder: a comprehensive assessment using the theory of mind assessment scale. Psychiatry Res. 272, 609–617. doi: 10.1016/j.psychres.2018.12.102 30616131

[ref21] Dennett D. C. (1978). Beliefs about beliefs. Behav. Brain Sci. 1, 568–570. doi: 10.1017/S0140525X00076664

[ref22] Francesconi M. Minichino A. Carrión R. E. Chiaie R. D. Bevilacqua A. Parisi M.. (2016). Theory of mind as a mediator variable between neurocognition and functioning in young individuals in treatment with secondary services for non-psychotic disorders. Psychiatry Res. 246, 415–420. doi: 10.1016/J.PSYCHRES.2016.09.057, PMID: 27788462

[ref23] Gabbatore I. Dindar K. Pirinen V. Vähänikkilä H. Mämmelä L. Kotila A.. (2023). Silent Finns and talkative Italians? An investigation of communicative differences and similarities as perceived by parents in typically developing children. First Lang. 43, 313–335. doi: 10.1177/01427237221149310

[ref24] Gandolphe M. C. Lecluyse B. Triquet C. Brunelle E. Duparcq J. P. Nandrino J. L. (2018). Mind reading abilities in opiate-dependent patients: an exploratory study. Compr. Psychiatry 83, 46–52. doi: 10.1016/J.COMPPSYCH.2018.03.001, PMID: 29562165

[ref25] Grover V. (2015). Theory of mind: concept and application for classroom learning. Eur. Acad. Res. II, 13050–13060.

[ref26] Happé F. G. E. (1994). An advanced test of theory of mind: understanding of story characters’ thoughts and feelings by able autistic, mentally handicapped, and normal children and adults. J. Autism Dev. Disord. 24, 129–154. doi: 10.1007/BF02172093, PMID: 8040158

[ref27] Happé F. Frith U. (1996). The neuropsychology of autism. Brain 119, 1377–1400. doi: 10.1093/BRAIN/119.4.1377, PMID: 8813299

[ref28] Karmakar A. Dogra A. K. (2019). Assessment of theory of mind in adults: beyond false belief tasks. Act Nerv Super 61, 142–146. doi: 10.1007/S41470-019-00028-1

[ref29] Kulke L. Wübker M. Rakoczy H. (2019). Is implicit theory of mind real but hard to detect? Testing adults with different stimulus materials. R. Soc. Open Sci. 6:190068. doi: 10.1098/rsos.190068, PMID: 31417713 PMC6689622

[ref30] Laghi F. Cotugno A. Cecere F. Sirolli A. Palazzoni D. Bosco F. M. (2014). An exploratory assessment of theory of mind and psychological impairment in patients with bulimia nervosa. Br. J. Psychol. 105, 509–523. doi: 10.1111/bjop.12054, PMID: 24117350

[ref31] Laghi F. Terrinoni A. Cerutti R. Fantini F. Galosi S. Ferrara M.. (2016). Theory of mind in non-suicidal self-injury (NSSI) adolescents. Conscious. Cogn. 43, 38–47. doi: 10.1016/J.CONCOG.2016.05.004, PMID: 27236355

[ref32] Lecce S. Devine R. T. (2022). Theory of mind at school: academic outcomes and the influence of the school context. Infant Child Dev. 31:e2274. doi: 10.1002/ICD.2274

[ref33] Massaro D. Castelli I. (2009). Mentalization in communicative and socio-relational interactions: considerations about a theory-of-mind modelling. Stu. Commun. Sci. 9, 103–130.

[ref34] Matthews D. Biney H. Abbot-Smith K. (2018). Individual differences in Children’s pragmatic ability: a review of associations with formal language, social cognition, and executive functions. Lang. Learn. Dev. 14, 186–223. doi: 10.1080/15475441.2018.1455584

[ref35] Onishi K. H. Baillargeon R. (2005). Do 15-month-old infants understand false beliefs? Science 1979, 255–258. doi: 10.1126/SCIENCE.1107621 PMC3357322 15821091

[ref36] Ouellet J. Scherzer P. B. Rouleau I. Métras P. Bertrand-gauvin C. Djerroud N.. (2010). Assessment of social cognition in patients with multiple sclerosis. J. Int. Neuropsychol. Soc. 16, 287–296. doi: 10.1017/S1355617709991329, PMID: 20167136

[ref37] Poulin-Dubois D. Goldman E. J. Meltzer A. Psaradellis E. (2023). Discontinuity from implicit to explicit theory of mind from infancy to preschool age. Cogn. Dev. 65:101273. doi: 10.1016/J.COGDEV.2022.101273

[ref38] Premack D. Woodruff G. (1978). Does the chimpanzee have a theory of mind? Behav. Brain Sci. 1, 515–526. doi: 10.1017/S0140525X00076512

[ref39] Romozzi M. Di Tella S. Rollo E. Quintieri P. Silveri M. C. Vollono C.. (2022). Theory of mind in migraine and medication-overuse headache: a cross-sectional study. Front. Neurol. 13:968111. doi: 10.3389/fneur.2022.968111, PMID: 36119667 PMC9479534

[ref40] Schneider D. Slaughter V. P. Dux P. E. (2017). Current evidence for automatic theory of mind processing in adults. Cognition 162, 27–31. doi: 10.1016/j.cognition.2017.01.018, PMID: 28189035

[ref41] Skinner B. (1938). The behavior of organisms: An experimental analysis. New York, NY: Appleton-Century.

[ref42] Smogorzewska J. Szumski G. Grygiel P. (2020). Theory of mind goes to school: does educational environment influence the development of theory of mind in middle childhood? PLoS One 15:e0237524. doi: 10.1371/journal.pone.0237524, PMID: 32797114 PMC7428351

[ref43] Stone V. E. Baron-Cohen S. Knight R. T. (1998). Frontal lobe contributions to theory of mind. J. Cogn. Neurosci. 10, 640–656. doi: 10.1162/089892998562942, PMID: 9802997

[ref44] Tirassa M. (1999). Communicative competence and the architecture of the mind/brain. Brain Lang. 68, 419–441. doi: 10.1006/BRLN.1999.2121, PMID: 10441187

[ref45] Tirassa M. Bosco F. M. (2008). “On the nature and role of Intersubjectivity in human communication” in Enacting Intersubjectivity: A cognitive and social perspective on the study of interactions. eds. Morganti F. Carassa A. Rova G. (Amsterdam, NE: IOS Press), 81–95.

[ref46] Tirassa M. Bosco F. M. Colle L. (2006a). Rethinking the ontogeny of mindreading. Conscious. Cogn. 15, 197–217. doi: 10.1016/J.CONCOG.2005.06.005, PMID: 16099174

[ref47] Tirassa M. Bosco F. M. Colle L. (2006b). Sharedness and privateness in human early social life. Cogn. Syst. Res. 7, 128–139. doi: 10.1016/J.COGSYS.2006.01.002

[ref48] Trevarthen C. (1998). “The concept and foundations of infant intersubjectivity” in Intersubjective communication and emotion in early ontogeny. ed. Bråten S. (Cambridge: Cambridge University Press), 15–46.

[ref49] Wang Z. (2015). Theory of mind and children’s understanding of teaching and learning during early childhood. Cogent Educ. 2:1011973. doi: 10.1080/2331186X.2015.1011973

[ref50] Watson J. B. (1913). Psychology as the behaviorist views it. Psychol. Rev. 20, 158–177. doi: 10.1037/h0074428

[ref51] Wellman H. M. Cross D. Watson J. (2001). Meta-analysis of theory-of-mind development: the truth about false belief. Child Dev. 72, 655–684. doi: 10.1111/1467-8624.00304, PMID: 11405571

[ref52] Wimmer H. Perner J. (1983). Beliefs about beliefs: representation and constraining function of wrong beliefs in young children’s understanding of deception. Cognition 13, 103–128. doi: 10.1016/0010-0277(83)90004-5, PMID: 6681741

